# A Focus Group Study to Understand the Perspectives of Physiotherapists on Barriers and Facilitators to Advancing Rehabilitation in Low-Resource and Conflict Settings

**DOI:** 10.3390/ijerph182212020

**Published:** 2021-11-16

**Authors:** Cornelia Anne Barth, Maggie Donovan-Hall, Catherine Blake, Noor Jahan Akhtar, Joseph Martial Capo-Chichi, Cliona O’Sullivan

**Affiliations:** 1 School of Public Health, Physiotherapy and Sports Science, University College Dublin (UCD), Belfield, D04 V1W8 Dublin, Ireland; c.blake@ucd.ie (C.B.); cliona.osullivan@ucd.ie (C.O.); 2 Cochrane Switzerland, Centre for Primary Care and Public Health (Unisanté), University of Lausanne, 1010 Lausanne, Switzerland; 3 School of Health Sciences, Faculty of Life and Environmental Sciences, University of Southampton, Southampton SO17 1BJ, UK; mh699@soton.ac.uk; 4 Bangladesh Health Professions Institute, University of Dhaka, Dhaka 1343, Bangladesh; shapla.physio@gmail.com; 5 Centre de Dépistage et de Traitement de L’ulcère de Buruli d’Allada, Ministry of Health, Allada BP 03, Benin; cjosephmartial@gmail.com

**Keywords:** rehabilitation, physiotherapy, health system, low resources, conflict, qualitative research

## Abstract

Physiotherapy as a health profession is continuously evolving in high-income countries (HIC). The highest burden of disease globally, however, is in low-resource and conflict contexts (LR-CC), resulting in unmet rehabilitation needs. Rehabilitation service models from HIC often face challenges when applied to the fragile health systems of LR-CC. It is important to engage rehabilitation experts living and working in LR-CC to guide service development. This study aims to understand physiotherapists’ views and perspectives of current rehabilitation services, of how these services can be strengthened over the next 10 years and of the role of physiotherapy within this development. Focus group discussions (FGDs) were conducted with 31 physiotherapists from 18 LR-CC using English as a common language. Audio recordings were transcribed verbatim. Data analysis was guided by thematic analysis. Participants provided deep insights into the complexity of developing rehabilitation services within fragile health systems. Participants agreed that physiotherapy lacked recognition and resources to be utilised effectively. Interacting themes as crucial prerequisites for strengthening the sector included (1) significance of context, (2) professional identity, and (3) professionalisation supported by workforce development and advocacy. These results are an important evidence base for informing the development of rehabilitation programmes in LR-CC and for future research.

## 1. Introduction

Physiotherapy as a health profession is continuously evolving in well-resourced and high-income countries (HIC). This has led to a growing body of research exploring topics such as advanced practice [1], direct access [2], best practice [3,4,5], and cost-effectiveness [6,7,8,9,10]. The highest burden of disease globally, however, is in low-resource and conflict contexts (LR-CC), resulting in enormous rehabilitation needs [11,12]. Service models from HIC applied to these contexts often struggle in adjusting to the specificities and complexities of LR-CC [13]. The number of physiotherapists and rehabilitation specialists is alarmingly low [14,15,16,17,18,19] and quality research is rare [20,21,22,23]. Scarce workforce leads to many physiotherapists operating on their own or in small teams with little opportunity for professional exchange, training, and career development. Furthermore, their clinical responsibilities and duties may vary and range from acute care to late rehabilitation across multiple medical specialties with limited equipment or infrastructure [24,25,26,27]. The populations requiring rehabilitation often lack access and awareness or attend services in advanced stages of their health condition complicating the outcome and success of rehabilitation interventions [28,29]. In challenging settings of high need and low supply, international organisations alongside local actors and initiatives can be an indispensable backbone of fragile systems where they support rehabilitation projects and training of rehabilitation professionals [30,31,32,33]. Physiotherapists often have a leadership role in developing the rehabilitation sector in LR-CC [25]. As the only rehabilitation professional present in some settings, they may fulfil a broad range of rehabilitation activities, which in HIC would be covered by multidisciplinary teams [22]. Individuals providing physiotherapy in such settings have valuable insight and knowledge into specific contextual challenges and potentially far-reaching impact on developing the rehabilitation sector there. It is crucial to inform decisions on programming and strengthening rehabilitation services in fragile, low-resource health systems by consulting rehabilitation experts living and working in these contexts. Therefore, the aims of this study were to understand physiotherapists’ views and perspectives of current rehabilitation services and how these services can be strengthened over the next 10 years, aligned to the WHO’s Rehab 2030 initiative, and the role of physiotherapy within this development [34].

## 2. Materials and Methods

### 2.1. Study Design

A qualitative study design was chosen using focus group discussions (FGDs) to meet the aims of this study and generate understanding and knowledge about how rehabilitation services can be developed over the next 10 years. The approach was pragmatic, inductive and guided by thematic analysis [35,36,37]. This study is reported following “Standards for Reporting Qualitative Research” (SRQR) guidelines [38].

### 2.2. Setting

Five FGDs were carried out directly after the Congress of the World Confederation for Physical Therapy (WCPT; now: World Physiotherapy) 2019 in Geneva [39]. Congress and FGD attendance of the participants was financed by the International Committee of the Red Cross (ICRC) that runs or supports rehabilitation projects in 35 countries worldwide including capacity building of rehabilitation workforce [30].

### 2.3. FGD Facilitators’ Profiles

The first author (C.A.B.) and FGD main facilitator was the ICRC global rehabilitation/physiotherapy advisor at the time of study. She has extensive experience in working in settings where participants come from and knew approximately half of the participants personally from previous collaborations. All five FGD facilitators were physiotherapists by profession. Two facilitators were ICRC technical supervisors and two, including co-authors C.O. and C.B., were researchers.

### 2.4. Study Population

ICRC-employed or ICRC-partner physiotherapists could apply for funding of congress attendance if they actively contributed to and presented at the congress. Sponsored physiotherapists were invited to the post-congress FGDs. Participation was not an automatic consequence of congress attendance, but entirely voluntary, free of charge and included provision of food and accommodation. This pre-selection provided a certain level of professional commitment, skill and English proficiency, geographical and cultural diversity as well as the opportunity to gather key professionals of LR-CC around a table. All interested applicants were included. Thirty-one physiotherapists agreed to participate, enabling five FGDs to be conducted. Each FGD consisted of five to seven participants and one facilitator. Using the list of participants, group allocation was deliberate, aiming at diversity in sex, cultural background, language, age, professional training, experience, and role. All represented countries, apart from Switzerland, classify as low-resource settings [40], certain countries or contexts within countries classify additionally as conflict settings, for example protracted crises with armed clashes flaring up regularly, over extended periods of time, refugee or post-conflict settings [41,42]. While countries can experience multiple and changing contexts, Table 1 describes the principle context at the time of study.

### 2.5. Ethics Statement

Prior to the FGDs, all participants gave written informed consent and completed a demographic questionnaire, summarised in Table 1. They were free to withdraw from this study at any moment. The study protocol was approved by the ICRC physical rehabilitation programme, the ICRC data protection office and the Ethics Committee of the canton of Geneva, Switzerland (Reference Req-2019-00027).

### 2.6. Data Collection

FGD guiding questions were developed by C.A.B. and C.O. and included several consultation rounds with the other facilitators. The FGD format followed Halcomb et al.’s guidelines for research with culturally and linguistically diverse groups (Table 2) [43].

Where language barriers led to inability of participants to express themselves, they were free to speak their preferred language such as French, Arabic, or Khmer. Usually there was another participant who spoke the same language and assisted with translating to English. If participants chose to contribute in English, facilitators were instructed to give time and language support so that everyone had a chance to formulate their contributions. Facilitators aimed to address potential influences of cultural, gender, age, seniority, or rhetoric disparities. It was deemed crucial to moderate the FGDs with fairness and empathy so that everyone felt safe and got an equal chance to contribute [43]. The FGDs lasted between 90 and 105 min, with coffee breaks separating the collective sessions from the FGDs (Table 2). The audio recordings were carried out on smartphone Dictaphone, saved on password protected computers after the FGDs and deleted from all smartphones.

### 2.7. Data Analysis

One female physiotherapist from Bangladesh (N.J.A.) and one male physiotherapist from Benin (J.M.C.-C.) were invited as collaborators. N.J.A. and J.M.C.-C. were not FGD participants but representative of invited participants. This permitted a more diverse, globally representative team to gain first-hand insight from co-researchers with lived experience and to enhance credibility and trustworthiness of the data analysis.

FGDs were first transcribed verbatim by two FGD participants and a research assistant. C.A.B., who was familiar with various accents and specific expressions, reread and corrected each transcript.

Participants often portrayed themselves and each other with clearly identifiable descriptions. Despite deidentification of all personal names, the transcripts could not be anonymised sufficiently without altering contextual understanding. For this matter, only the primary researchers C.A.B. and C.O. had access to the raw data. C.A.B. independently coded all five FGDs, C.O. independently coded two of the five FGDs. Data were analysed using an inductive approach of thematic analysis and following the stages outlined by Braun and Clarke [36,44]. C.A.B. first read all transcripts making notes, before reading them again and starting initial coding in NVivo, version 12.6.1 (QSR International, Burlington, MA, USA). The analysis was carried out systematically across groups and independently of the questions. This stage generated a high number of loose, low-level codes and a large volume of potential quotes (around 800) where names of persons, health or education institutions and geographical locations were sufficiently deidentified to allow data protection. Microsoft Excel, version 16.52, was used at this stage and the material was shared with the other authors for coding on their side. Simultaneously, C.A.B. started clustering codes into potential themes and sub-themes using NVivo mind maps and other visualising software (Microsoft PowerPoint, version 16.52). In an ongoing, iterative process of online exchange, codes and themes were discussed among all researchers and critically compared to the early thematic model developed by C.A.B. to mitigate any bias that may occur when interpreting the data.

C.A.B. reread transcripts repeatedly for additional codes and quotes, merging and refining as the analysis developed. The heterogeneous background of researchers allowed rich discussions on the analysis. This process of verification was continued until agreement on the final themes was reached.

## 3. Results

Participants were 31 physiotherapists including three international physiotherapists deployed in humanitarian assignments outside their home country. All were part of a global physiotherapy workforce and either employed by (*n* = 17) or connected to the ICRC (*n* = 14) through formal partnerships in countries where the ICRC supports rehabilitation projects. Active language skills varied widely. Speaking time of participants, although balanced by facilitators, correlated with English skill and personality, probably also with perceived norms on hierarchy, culture, and gender.

To maintain anonymity and avoid recognition of individuals working in very specific contexts or roles (i.e., in some contexts there is only one physiotherapist), none of the quotes have been labelled with any participant characteristics.

### 3.1. Three Key Themes: Context, Professional Identity and Professionalisation

**Figure 1:**
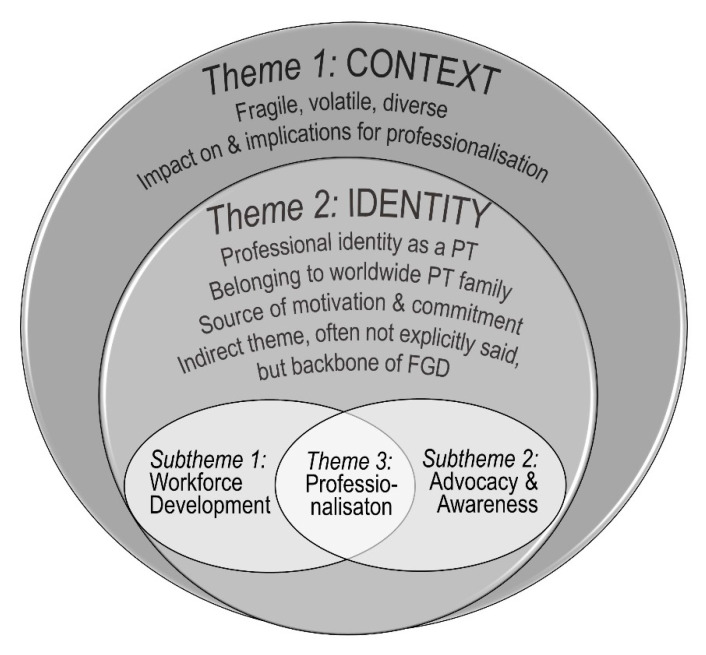
**Themes.** The main themes are illustrated in the circles. The themes ‘Context’ and ‘Professional Identity’ frame, define and influence the sector-specific theme ‘Professionalisation’, which comprises the sub-themes ‘Workforce Development’ and ‘Advocacy and Awareness Raising’. Figure 2 zooms further into these two subthemes. PT = physiotherapist/physiotherapy; FGD = focus group discussions.

**Figure 2:**
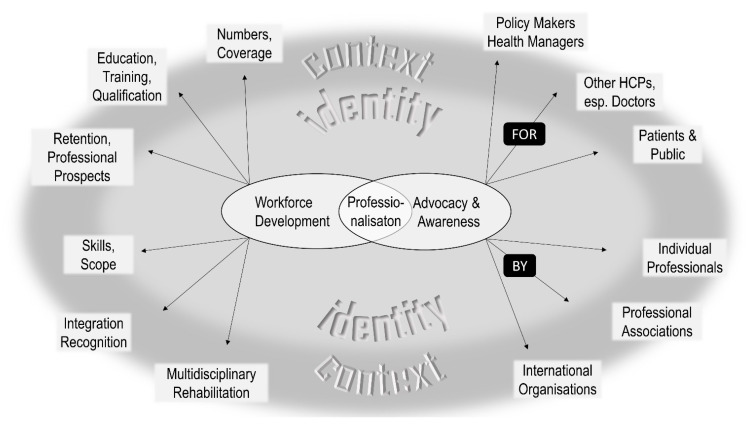
**Subthemes and Elements.** The key subthemes under the theme ‘Professionalisation’ were ‘Workforce Development’ and ‘Advocacy and Awareness Raising’. These two subthemes contain further divisions into 6 elements each. HCPs = health care professionals.

Three key themes were identified and are illustrated in Figure 1. As participants worked across and came from a wide range of different countries, ‘Context’ is presented as theme 1 to underpin how these different environments influenced the FGDs. The term ‘Professional Identity’ describing theme 2 reflected an underlying current throughout the FGDs. Both themes 1 and 2 were found to envelope and impact on the rehabilitation-specific theme 3 ‘Professionalisation’. This theme was the most elaborated and emerged from contributions describing the lack of and need for professionalisation. Two subthemes were identified as fundamental and directional to this regard: ‘Workforce Development’ and ‘Advocacy and Awareness Raising’, each separated into six further elements (Figure 2).

### 3.2. Theme 1: Context

The participants represented a great diversity of contexts (Table 1). The given circumstances of their working environment framed all statements. Accordingly, this defined the steps towards rehabilitation strengthening.

War, volatility, poverty, and corruption were recurring discussion points of participants representing countries of prolonged conflict or post-conflict instability. Interruption of services seemed a frequent experience for participants from such contexts. Some participants described how access to and for the population was hampered by tense security and military checkpoints, in addition to the usual access barriers of low-resource settings. Professional development could be directly linked to impactful political decisions such as a ceasefire.

“*Now we are in peace in the country. So, we can do something and not only surviving but to do something to our profession*.”

Participants from countries hosting refugees from neighbouring conflicts described how health systems may get overloaded and fragmented.

“*The hospital project is for weapon wounded. … we have the physiotherapy, the normal private sector and we have … the ICRC who is supporting weapon wounded and disabled people and the vulnerable … from [neighbouring country at war] … they are very, very, very vulnerable people … so it is two different sectors*.”

Some participants elaborated on their countries’ significant urban/rural discrepancies (subtheme 1a). Other contributions included descriptions of poor, almost non-existent rehabilitation sector development (subthemes 1d, 1e and 2) and how the absence of an infrastructure for knowledge transfer influenced their professional development.

“*These things do not reach us, the articles or the practice of physiotherapy. So we need these things to be available … but mostly the internet in some locations [is] not available, you know access is difficult to the network … where I come from, even a telephone call you cannot make*.”

One participant described a situation that could be attributed to low health literacy, access challenges and cultural aspects, which in combination may cause and exacerbate certain health conditions.

“*[The] mother of that patient doesn’t know about the … pregnancy … low age they get married and … they deliver at home and because of that the level of cerebral palsy is high*.”

Participants valued and resonated with the similar experiences shared by fellow physiotherapists. They discussed how the challenges within complex contexts had helped educate them broadly about health systems, politics and about performing under adverse conditions.

“*You are already … living daily with a lot of tension and you are still continuing … and working a lot. This give[s] you more opportunity to have well experience. … When you are saying that [colleagues from HIC] are using … shockwave … or ultrasound … I don’t have electricity … And you are … going to treat the persons which should … be at the ICU [intensive care unit] … these challenges give you a lot of experience*.”

### 3.3. Theme 2: Professional Identity

Professional identity and the feeling of belonging to a global physiotherapy family, reinforced during the congress, seemed to have created a strong sense of motivation, purpose and identity-building. It emerged that a number of physiotherapists had never met colleagues outside their workplaces before due to lack of means or a tense security situation that had compromised travel options.

“*When you work a lot in a context and the things don’t improve, you have no results and you feel a bit frustrated and when you … discover that many colleagues in different countries, different contexts have same challenges as you even bigge[r] challenges than you … and you can learn from their experiences and try to improve your context*.”

As much as participants felt they ‘belonged’ on a global level, this was countered by descriptions of their almost invisible professional identity on a national level. Many discussions revolved around a profound lack of recognition and awareness (subthemes 1d and 2).

### 3.4. Theme 3: Professionalisation

#### 3.4.1. Subtheme 1: Workforce Development

According to participants, rehabilitation strengthening depended on the following elements of adequate quantity and distribution of professionals (a), who are qualified (b), skilled and broadly positioned (c), integrated and recognised (d), with career prospects (e), and who represent multiple professions within the rehabilitation sector (f).

##### (a) Numbers, Coverage

In the studied contexts, the physiotherapy workforce was often so small that some participants could precisely list numbers and locations of their fellow colleagues in countries oftentimes as vast as Western Europe. Already scarce services were often concentrated in the metropoles, resulting in zero access for the rural population.

“*For a population of two million people there is only five physiotherapists … of these … four are working in the only rehabilitation centre that exists in the country, it is in the capital. And the other physiotherapist work[s] in the [capital’s] public hospital … to reach from the capital to the most far regions maybe it is nine hours travel with difficult roads … [and] two days to come from the most far islands to the capital*.”

Even in countries where the physiotherapy sector could be considered relatively well developed, there was an alarming shortage and poor distribution of services. A large part of the workforce seemed to be placed in urban private services or aid organisations.

##### (b) Education, Training, Qualification

Physiotherapy training programmes were described as being insufficient with regard to quantity, location and sustainability of existing courses and to their affordability and acceptability. If entry level education was being offered, the number of graduates was far too low to meet rehabilitation needs. Education was often viewed as either too expensive without scholarships or not attractive in a population where the profession remained unknown or misunderstood.

“*Even for me, when I went to school to study it, some people were saying: ‘There is no better qualification that you[’re] suppose[d] to study? Why do you study physiotherapy? What is the benefit of that?’ So people were blocking my interest even to do it*.”

Some participants raised concerns about the low quality or absence of clinical education and the fact that graduated students entered the job market lacking clinical skills. Participants who were teaching or supervising new graduates in their workplaces, provided examples of a low capacity of teachers, including themselves. Options for teachers’ continuing professional development (CPD) were scarce and teaching methods outdated.

Besides degree-upgrading opportunities, participants suggested offering teacher trainings in leadership, teaching methods and clinical teaching. They repeatedly discussed the need to develop curricula to meet international standards. This was contrasted by examples for which international standards were of limited use and interfered with contextual realities.

Where entry level education is insufficient and the number of graduated physiotherapists too low to cover needs, CPD or any form of postgraduate training was discussed as being an even greater challenge for participants.

“*They have to go out of [the country] and pay lots of money … that’s why we have no opportunity to upgrade our knowledge*.”

Although further studying for advanced degrees was a key ambition of many participants, some acknowledged the need of a broad-based, widespread workforce to allow a representation at all levels, including community based rehabilitation.

“*We are currently discussing with Ministry of Health and Ministry of Education to include diploma physiotherapy in the curriculum … because we need them at the low level of care*.”

It was discussed how professional associations and international organisations play an important role in implementing CPD and a number of participants were involved in it.

Others stated that the lack of postgraduate education cost the profession its credibility within health systems and among established health care professionals or policy makers. The statements highlighted the participants’ thirst for more knowledge and skills as well as a need for recognition, awareness, and health systems integration for the sector in general (see subthemes 1d and 2).

“*If you want the doctor or the other health professional to recognise the physical therapy you need to upscale the degree. Because right now when we talk the doctor, doctor said your level cannot talk with us. That’s the problem and then we do not have any physiotherapists position in the ministry*.”

##### (c) Skills, Scope

Despite the perceived inferiority, participants proved to be key players for rehabilitation in their countries with competencies that went well beyond clinical ones, for example leadership skills for high-level stakeholder meetings with government officials. A participant from a country with better integrated rehabilitation could report of physiotherapists’ success in this development. Such examples were taken up by other participants as models, encouragement, and opportunities for future exchange.

*“When I graduate[d], we went to the ministry of health and we said we would like to work in hospital setup where there is orthopaedic surgeon … neurologist and neurosurgeon … And they said no. For this country it is not priority. That was ten years ago, but now the ministry is asking the association to develop curriculum, strategies and policies …they have asked the association to produce a road map for physiotherapy professionals for the next ten years, on [the] number of professionals needed for primary, secondary, tertiary hospital[s] and specialized hospitals … and which levels, doctors or masters, BSc holders. So, the opportunity is there now.”*

Many participants disapproved the labels they were confronted with by health care professionals or patients, who associated physiotherapy with delivery of technical skills only such as massage or the execution of exercises prescribed by a medical doctor. Some stated they were facing obstacles when trying to apply patient-centred physiotherapy based on clinical reasoning. Participants wanted physiotherapists to be found across the continuum of care and in influential, non-clinical positions. They expressed that a broad scope would help them succeed in strengthening the sector. Being aware of the increasing needs, especially related to management of non-communicable diseases (NCDs), participants were urging for more involvement and full-fledged integration into health systems.

##### (d) Integration, Recognition

Several participants acknowledged that improvements in education and training alone will not strengthen rehabilitation services if the underlying health systems were under resourced and poorly organised. In many countries, rehabilitation was associated with disability care under ministries of social affairs rather than recognised as a health domain.

Some participants discussed tensions between physiotherapy and medical professions where hierarchical structures were viewed as impacting on the delivery of services. The dependence on medical doctor’s prescription was perceived as degrading and incapacitating. Several participants reported a lack of autonomy and professional recognition being a significant barrier to developing services and professional practice.

“*Physio as a profession within the health is growing slowly but yet we have a lot of obstacles with medical doctors and physicians who are trying to put blocks in front of us*.”

##### (e) Retention, Professional Prospects

Low awareness and acceptability of physiotherapy and rehabilitation made it difficult to attract students to physiotherapy programmes. Here, participants envisaged opportunities and roles for themselves to promote the sector.

“*We have very rare of students to enrolment … very few students … we can make the awareness to … the high school students … so that they will know about the benefit of physiotherapy so they can consider that profession*.”

Owing to a lack of integration and awareness, participants reported of graduated and employed physiotherapists who do not get to work in their profession.

“*Physiotherapy is recruited by the Ministry of Health to work at the hospital but they do not work as a physiotherapy profession they work as the nurses, pharmacists and … administrator*.”

Staff retention in-country was reported as a challenge by some participants. The lack of professional recognition and progress leads to emigration and career change, despite the presence of established training programmes.

“*So we are graduating … [an] army of physios but most of them, they are leaving … to gulf countries or Europe for better life conditions and good opportunities … In a governmental hospital you will be supervised by a doctor … you will be just an applier. You will get a premade treatment plan and … repeat the same thing for one or two years … so they do not feel that they are developing in their career…. That’s why recently, there is a new fashion happening that people … after they finish their bachelor they go and study again medicine. … after wasting four years because at the end … you will always feel like … I am not recognised by the health profession*.”

##### (f) Multidisciplinary Rehabilitation

In several places, participants discussed how other important rehabilitation professions were missing besides physiotherapy.

“*Right now we do not have any OT [occupational therapist]. We don’t have ST [speech therapist]. So the physiotherapy in [country] [is] doing all the job of the OT and ST*.”

Participants stressed the need of a multidisciplinary workforce including other rehabilitation professions, both to cover needs and to increase leverage.

“*You have other professional rehabilitation as OT, no?”--“In my country we have … speech therapist … but they are … not more than five.”--“I ask this because our … strategy with … government … we have a federation of professional rehabilitation and we have [to] go together in the lobbying … because the matter of the profession is not only PT [physiotherapy], it is about rehabilitation*.”

#### 3.4.2. Subtheme 2: Professional Advocacy and Awareness Raising

Advocacy and awareness raising were deemed a key requirement towards rehabilitation strengthening within health systems. Most participants had extensive practice with it. Advocacy was done by professionals individually, by professional associations, by and with support of international organisations like the WCPT/World Physiotherapy and the ICRC. These were found crucial for their leverage and proficiency. Participants identified three target groups: policy makers and health managers, other health care professionals (especially doctors), patients and public. They are not separated into paragraphs as participants often named them in one response.

“*The first thing in my country is the lack of awareness at all levels. When I say at all levels, I mean at community level, health professionals about physiotherapy and at ministry of health level*.”

An international physiotherapist deployed abroad learned that multifaceted advocacy was indispensable in LR-CC.

“*The discovery for me was the importance … [of] advocacy of the profession and the association … I understood why it is important [to] repeat again and again … what is the competences of physiotherapists and how we can contribute, not only for the care of the patient, but also to the overall health system, how we can help other colleagues and how is important to have a national association to have a dialogue with policy makers and international organisations and have the support to develop the physiotherapy in the country*.”

The rationale was obvious as was the contrast to established professions in the health system. The lack of professionalisation resulted in incomprehension among patients, other health care professionals and decision makers.

“*Until you get disabled, or you have a pain or you need to go to a physio clinic, then you will understand what is physiotherapy. It is not like a dermatologist or gynaecologist or whatever of medical professions. They [the patients] know exactly what they [other medical professions] do even if you are not in need for them, in general*.”

Discussions around doctors issuing physiotherapy prescriptions revealed some participants’ concern that the profession was not understood well enough to be validly put under the doctors’ responsibility. Physiotherapy required repeated awareness raising, not only among the public, but in particular among doctors as the hierarchically superior profession whom they depended on. Participants argued that advocacy would not only lead to enhanced understanding by other health care professionals, but also to multi-professional interaction, and, eventually, to doctor’s referral rather than prescription.

The value of a professional body was key to promoting the profession. Associations had a role in advocacy for the target population, other health care professionals and decision makers, but also in bringing physiotherapists together. The FGDs reflected how the exchanges at the congress had increased participants’ understanding of professional associations, which were young and under resourced in most contexts.

“*I started to realise the importance of the … national associations of physiotherapy and how much its strengthening … [means] for the physios in their country. How they can be united. How they can share the ideas and give the force to be recognized nationally and later internationally. And this is what we are missing in [country]. The weakness of the association. It was just recently started*.”

Some participants were aware that adherence to the WCPT/World Physiotherapy provided them with additional duties and, thus, increased power when negotiating on government level.

“*We do … lobbying in our government because now … we are members of the WCPT, and we must follow the rules and the programmes*.”

Professional purpose and identity underlined the duty to promote the profession.

“*We need [to] show to ourselves and to the others that this profession is something … That it is a health profession that can change your life. So, it [is] for us and for the patient to believe in this*.”

Reports from participants from countries where physiotherapists had succeeded in establishing and obtaining influential roles in the health ministry were taken up with great interest by other participants.

In countries with low awareness of rehabilitation, some situations arise that at times may damage a (barely existing) reputation. For example, delayed uptake of services leads to poor outcomes, leading to a vicious cycle of poor awareness and recognition.

“*We received a lot [of] patients that tried another treatment and come to the physiotherapy as the last resort because nothing [else] have helped … they have many months or even years of evolution and possibilities of improvement are, of course, clinically very reduced*.”

It became clear from some participants’ contributions that the negotiations that accompanied each therapeutic intervention not only significantly reduced the actual treatment time, but also posed an ethical challenge.

“*Sometimes the situation can push you … out of the evidence … there are some patients…, they come with their own ideas: ‘If you treat me like [this] I am not going to accept.’ What you do then? You have to listen, then you … try and … convince him … favouring what he is saying, then almost leaving your line of profession, then later you come in agreement to treat him almost in the way that he [is] proposing, … less than what you want … as a professional*.”

Some participants stressed that advocacy came with a responsibility and required constant enhancement of service provision.

“*Now the role of physiotherapy there is to help identify this people …, give them awareness, also those who have reached to the hospital make proper assessment and give proper services to this people*.”

## 4. Discussion

### 4.1. The Role of Physiotherapists

The participating physiotherapists showed high levels of consensus pertaining to the topics discussed. This may have been the result of group dynamics and a feeling of unity within a disadvantaged profession. A common identity is a powerful resource. It seems crucial for the enormous task of strengthening rehabilitation in such contexts. The desired change is not going to come from the outside, but has to be initiated and sustained by a few. As ‘pioneers’ they need above-average motivation and commitment for this long-term goal. Many demonstrated that they have this leadership attitude.

The participants reported the breadth of skills required in these contexts, which are not part of a classical physiotherapy education. These competencies seem particularly pronounced in difficult, often highly politicised environments, and probably also derived from the participants’ roles in their countries, within international organisations and professional associations. However, these frontline workers cannot act on their own. Whilst their initiative is fundamental, they often lack positions that would allow them to achieve change. Often their leadership attitude and acquired competencies were not recognised by health system leaders.

Physiotherapists depend on commitment and support from fellow rehabilitation workers. They need to be supported by colleagues from other disciplines, especially influential and appreciative medical doctors. All health care professionals should be educated on how a strong rehabilitation sector benefits the entire health system [10,19,45]. Current and future challenges in global health care can only be addressed on a multidisciplinary base [46,47]. Such changes would also decrease the physiotherapists’ ‘advocacy burden’ and lead to more efficient health interventions across disciplines.

### 4.2. Full Integration in Health Systems

Sector development starts with a critical number of human resources, yet our results showed that numbers alone may be misleading. Physiotherapy services, which are concentrated in urban, private settings, do not address the profound scarcity in the public sector and in communities. It is there where physiotherapy is needed in order to develop alongside other health care professionals and within society [12]. As long as appropriate rehabilitation service coverage is not promoted, chances for the sector to advance are impeded.

Various publications have lately addressed the importance of integrating rehabilitation within health systems [48,49,50,51]. The global needs for rehabilitation are well described [52]. Equally acknowledged is their increase in numbers and complexity [16], especially with regard to NCDs and the role of rehabilitation [19,53,54,55,56], and the mismatch of needs and resources [11,16,57,58,59]. Cost-effectiveness studies show the economic benefit of rehabilitation [10,60,61]. Experts across the globe agree on the urgency of the situation, yet LR-CC struggle with so many challenges simultaneously. A starting point could involve investing in staff retention and using existing resources by offering qualified physiotherapists appropriate posts, implementing obligatory rotations including posts in rural areas and using graduated professionals to recruit and mentor mid-level rehabilitation workers.

### 4.3. Multi-Stakeholder Engagement and Support

Strengthening rehabilitation requires joint efforts and resources that may not or only partially be provided by a country’s government. Many suggestions for sector development are only possible with international support including scholarships [30,62], large development programmes for health and education systems, or innovative financing models [63]. International organisations, in partnership with local organisations and initiatives, play a fundamental role as they often sustain, substitute and support systems in the absence of these, namely in countries of great poverty or conflict [41,64]. Such partnerships are usually based on considerable economic dependencies and cultural differences. Ongoing media discussions on the decolonisation of aid reveal how delicate such operations are [65,66]. It is crucial to follow recommendations what were developed by all parties for effective multi-stakeholder collaborations [67,68]. Actors involved in the sector range from grassroots initiatives on the ground, non-governmental organisations (NGOs), disabled persons’ organisations to national and international professional associations, international organisations, universities from HIC and LR-CC [69,70]. The contextual expertise of local actors (including rehabilitation workers and patients) should be considered as a resource that is of equal importance as the financial means of big global players. A genuine interest in participatory approaches is key for the outcome of such collaborations.

### 4.4. The Importance of Context and Its Implications

The included participants represented 18 different countries and sometimes more than one context in the same country. The diversity of the represented contexts range from low-resource countries with relative political stability [71] to highly volatile armed conflict [72]. Recommendations on rehabilitation strengthening have to take the complexity and interplay of contextual realities into account, which are defined by the politics, history, economy, demographics, culture and climate of a country [12,19].

Countries of protracted crisis may at times get submerged with aid, but not have the adequate workforce due to long-term educational challenges and brain drain [73,74]. Such situations bring resources in form of international workforce, which more ‘neglected’ low-resource countries may not have. However, these may dry up in the crisis contexts as soon as international organisations withdraw [75,76]. Any sustainable development there is highly challenging. The benefit of rehabilitation in such emergencies, though, may make a huge difference for weapon-wounded individuals and prevent or alleviate disabilities [31,77]. This can contribute to awareness raising and set the ground for future steps in sector strengthening.

In less volatile low-resource countries it may help to seek regional collaborations with comparable, more advanced countries [25,78], as stated in the strategic objectives of the WCPT Africa Region [79].

The implications of our study go beyond LR-CCs. They involve an entire sector in a global environment: it includes international organisations, mainly financed by HIC, and HIC-trained rehabilitation professionals deployed to LR-CC. It includes neighbouring or immigration countries where displaced populations end up presenting poly-traumatic, unfamiliar, complex conditions and where overloaded health systems have to deal with vulnerable populations. International support for the rehabilitation sector is important there, not only in conflict contexts [80].

The contributions from participants living in countries at war or too poor or unaware to develop a sector showed that rehabilitation workforce may have to be built outside these countries. Very challenging contexts are too unstable to deliver sustainable training programmes. Regional or global collaborations on training programmes and teacher exchange become increasingly important [70]. This includes countries with cultural similarities and relative stability, where rehabilitation workforce could be built [78] and where international investments are key.

Despite the diversity of contexts from which the participants come from, our findings demonstrate several key themes that are relevant across many LR-CC and provide guidance for the way forward for the development of rehabilitation in these contexts.

### 4.5. Limitations, Strengths and Trustworthiness

The setting of post-congress FGDs allowed to include only physiotherapists in this study. Contributions from other rehabilitation professionals could have been informing. Yet, a shared professional background was likely to reinforce a feeling of belonging, which we deemed important considering the huge diversity of participants.

The sampling strategy using a naturally occurring group meant that the researchers did not choose participants themselves through the use of inclusion or exclusion criteria. Having such a big and varied group of physiotherapists face to face around a table was a one-time opportunity, which seems even more precious and unique since the subsequent COVID-19 global pandemic. It would have been unfortunate not to seize that unique opportunity for an unfunded research project.

As ICRC staff, three facilitators had roles of former or future superiors, recruiters, or informal mentors of participants. All facilitators may have been perceived as more experienced by many participants and thus in an indirect role of authority. Considering dynamics of culture, gender and hierarchy in countries of origin and in humanitarian or academic institutions, it is likely that this setup has influenced FGD dynamics. It may have affected free expression of opinion and overstimulated self-consciousness or self-promotion. Additionally, the facilitators’ background and professional agenda may have influenced the direction the FGDs took. The facilitators were conscious about these risks and encouraged to openly address this during the encounters with participants. The facilitators’ connection to the participants as part of a global team, linked through the ICRC as an employer or partner, was considered highly important to reinforce teambuilding and to develop ideas about what roles might result from being part of such a team. Additionally, the ICRC’s function as a sponsor or an employer may have biased contributions. For example, participants discussed more about the ICRC than on the importance of national and local organisations and initiatives, known to be pillars for community-based rehabilitation, inclusion and informal professional trainings. Some participants may have responded differently had they been interviewed individually. However, we wanted to utilise the dynamics of FGD and harness the stimulating effect of the congress, for most participants a first-time experience, to consolidate exchange and learning. English as a foreign language means the used expressions corresponded with the language skills of the respective participant. Thus, a compromise on information content and a first interpretation (i.e., having to choose a less accurate term or having a contribution paraphrased or translated by another participant) may start the very moment a contribution is made. In addition, despite several rounds of transcript checking, recording quality, vocal volume and certain accents have compromised content retrieval of some passages. Nonetheless, the strength of the FGDs is that the use of a common language allowed participants from across the world to exchange face to face and in real time. Transferability is a particular strength of this study given the diversity of participants. Each in their roles as key actors represented an entire context. We are aware, though, that some of their contributions may not reflect the opinion of fellow physiotherapists in the respective countries. This study’s trustworthiness is enhanced through the participation of co-researchers from LR-CC themselves who had not participated in the FGDs, yet confirmed how the results matched with their reality. This strengthens our assumption that the results resonate with colleagues from different LR-CC and the recommendations are valid beyond the studied contexts.

## 5. Conclusions

Our study adds to global initiatives on rehabilitation strengthening as a unique multi-country qualitative research. It revealed details about what situations arise if rehabilitation lacks professionalisation, recognition and integration and what frontline workers concretely suggest to change that. Our results illustrate that simultaneous progress in the subthemes and their elements is necessary for the sector to advance. Such a process is continuous and should be informed by the different realities and perspectives in LR-CC. Non-action will have unaffordable consequences—from an economic perspective [10,81] as well as with regard to health equity [49,82], professional ethics [12], and sustainable development [55,83,84,85,86]. The consequences will be more directly seen in the countries themselves but have an impact on the future of global health.

Rehabilitation addresses the functioning of an individual “in interaction with their environment” [87], which will shape the functioning of the society they live in. Beyond an impact on individuals, the professionalisation of physiotherapy and rehabilitation is likely to also influence the functioning of society as a whole.

To our knowledge, this is the first study that has given physiotherapists from such a diversity of challenging contexts a voice. We need more such voices like that and more funded research with implicated rehabilitation professionals, including other disciplines in this field.

An increasingly globalised health sector is confronted with increasingly complex health challenges, exacerbated by the consequences recurring conflicts and climate change. A strong and integrated rehabilitation sector is a key element in addressing such challenges. Its development requires the collaboration of physiotherapists from high- and less resourced settings, health professionals, managers and policy makers, professional associations, small local and large international organisations—using the unique resources each has to offer to work toward the sustainable development goal of “Health for All”.

## Figures and Tables

**Table 1:** Participant profiles.

Demographics			
Sex	Female 13/Male 18 (42% female)		
Age range in years (median)	25–68 (34)		
Workplace			
African Region	Algeria, Benin, DR Congo, Ethiopia, Guinea-Bissau, Ivory Coast, Madagascar, Mali, Niger, South Sudan, Sudan, Togo		
Eastern Meditarranean Region	Afghanistan, Iraq, Lebanon		
European Region	Switzerland *		
South-East Asian Region	India, Myanmar		
Western Pacific Region	Cambodia		
Work Context (numbers of participants)			
High-income country (1)	Switzerland * (1)		
Low-resource context (7)	Benin (2), Guinea-Bissau (1), India (New Delhi) (1), Ivory Coast (1), Madagascar (1), Togo (1)		
Hosting refugees from neighbouring countries (3)	Algeria (1), Lebanon (2)		
Prolonged conflict (14)	Afghanistan (3), DR Congo (2), India (Kashmir) (1), Iraq (1), Mali (1), Myanmar (2), Niger (1), South Sudan (2), Sudan (1)		
Post-conflict (6)	Cambodia (4), Ethiopia (2)		
Profession		Female	Male
Experience	<5 Years 5–10 Years >10 Years	1 7 5	0 5 13
Physiotherapy training	Assistant Diploma BSc MSc (MPH) PhD	0 5 6 2 0	1 2 9 5 1
Current role	Physiotherapist (clinician) Physiotherapist (clinician+) ** Physiotherapist (team leader) Project assistant Project manager/Advisor Teacher/Trainer	6 3 1 1 2 0	9 2 3 1 1 2

* 1 international physiotherapist was temporarily based in Europe between two assignments and referring to previous field experiences in Africa and the Middle East during the FGDs. ** clinician AND trainer/team leader/project assistant.

**Table 2:** Guiding questions for FGD facilitators.

Topic	Time	Question
Ground rules	10 min	What should every one of us do or respect in a group discussion?
Warm up	5 min	Can you tell us your name, country and workplace?
Introductory question	15 min	Is anyone happy to share his or her experience at the congress?
Guiding questions	90–105 min	Based on experiences during the congress where do you see physiotherapy in your context in the next 10 years? What do you perceive as the priority health conditions that need to be addressed in your country and what is the role of physiotherapy in addressing these health priorities? What are the challenges and opportunities for the development of physiotherapy in your country? What is your role in addressing these challenges and harnessing opportunities?
Wrap-up	15 min	Of all the things we’ve just discussed, what would you say are the most important conclusions you will take with you?

## Data Availability

Data are not publicly available because the contexts are often sensitive and the number of physiotherapists in the countries included is small, so participants may be identifiable. Participants did not give consent for the data to be stored in a public repository.
